# Chemical Composition and Antiproliferative Activity of the Ethanolic Extract of *Cyperus articulatus* L. (Cyperaceae)

**DOI:** 10.3390/plants10102084

**Published:** 2021-10-01

**Authors:** Éden Bruno Sousa da Silva, Lauro Euclides Soares Barata, Michelly Rios Arévalo, Leda Quercia Vieira, Waldionê Castro, Ana Lúcia Tasca Gois Ruiz, Adriana Della Torre, Kelly Christina Ferreira Castro, Adilson Sartoratto, Leopoldo C. Baratto, Maxwell Barbosa de Santana, Antonio Humberto Hamad Minervino, Waldiney Pires Moraes

**Affiliations:** 1 Laboratory of Pharmacology, Institute of Public Health—(ISCO), Federal University of Western Pará (UFOPA), Santarém 68040-255, PA, Brazil; edenbrunoss@gmail.com (É.B.S.d.S.); barbosadesantana@gmail.com (M.B.d.S.); 2 P&DBIO-Laboratory of Bioactive Natural Products, Institute of Biodiversity and Forests (IBEF), Federal University of Western Pará (UFOPA), Santarém 68040-255, PA, Brazil; lauroesbarata@gmail.com (L.E.S.B.); michrios76@yahoo.com.br (M.R.A.); kelly_quimica@yahoo.com.br (K.C.F.C.); 3 Laboratory of Gnotobiology and Immunology (LAGI), Department of Biochemistry and Immunology, Institute of Biological Sciences (ICB), Federal University of Minas Gerais (UFMG), Belo Horizonte 31270-901, MG, Brazil; lqvieira@icb.ufmg.br (L.Q.V.); waldione@hotmail.com (W.C.); analucia@cpqba.unicamp.br (A.L.T.G.R.); AdrianaTorre@cpqba.unicamp.br (A.D.T.); 4 Laboratory of Pharmacognosy, Federal University of Rio de Janeiro (UFRJ), Rio de Janeiro 21941-599, RJ, Brazil; adilson@cpqba.unicamp.br (A.S.); leopoldo.ufopa@gmail.com (L.C.B.); 5 Laboratory of Animal Health, LARSANA, Federal University of Western Pará, UFOPA, Santarém 68040-255, PA, Brazil

**Keywords:** medicinal plant, cytotoxicity, arginase, antiproliferative, anticancer

## Abstract

*Cyperus articulatus* L. (Priprioca) is a plant of the Cyperaceae family traditionally used in traditional medicine in the Amazon region. Studies of the essential oil of this species have identified many terpene compounds. However, little is known about the possible uses of solid waste generated by the extraction of essential oils. This study aimed to investigate the chemical composition of volatile compounds and to evaluate the antiproliferative activity of the ethanolic extract of solid residues generated by the extraction of the essential oil of *C. articulatus* L. rizhomes in experimental models in vitro using peritoneal macrophages of mice and human tumor cell lines. The analysis of the chemical composition of volatile compounds indicated the presence of sesquiterpenes and particularly sequiterpenic ketones as main constituents. The results showed that the treatment with ethanolic extract of *C. articulatus* L. reduced the activity of the enzyme arginase and proliferation of cancer cells (*p* < 0.0001). The extract also showed no cytotoxicity in macrophages in concentrations between 12.5; 25 and 50 mg/mL (*p* < 0.0001). The results indicated that the extract of *C. articulatus* L. exerts antiproliferative activity (*p* < 0.0001) with low toxicity on healthy cells in experimental models in vitro.

## 1. Introduction

Traditional medicine is a cultural practice commonly employed in the Amazon region, relying on the use of medicinal herbs to treat diseases [1,2]. The Amazonian biodiversity comprises a huge reserve of biomolecules with pharmacological potential. In this sense, the search, characterization and isolation of biologically active substances are essential for the discovery of new drugs. Among biomolecules comprising the essential oils there are those with therapeutic potential which are already known for antimicrobial, anti-inflammatory, anticonvulsant, antitumor and anti-anxiety activities [3,4].

Cancer is a process linked to exacerbated cellular multiplication. Proliferation is mediated to biosynthesis of polyamines, glutamate, and proline, and are thus intrinsically related to the activity of enzymes that catalyze a production of these substances, such as arginase, which has an important role in cell proliferation and development of tumoral pathological processes [5]. Although progress has been made in chemotherapy treatments, many tumors remain difficult to treat, which induces the search for new antitumor agents. The search for antiproliferative medicines from medicinal plants led to the discovery of several compounds used by the pharmaceutical industry such as Taxol, isolated from *Taxus brevifolia*, vincristine, and vinblastine, isolated from *Catharantus roseus* and *Camptotheca acuminata*, all with anticancer activity [6].

Among the species of medicinal plants found in the Amazon region, we are particularly interested in *Cyperus articulatus* L., from the family Cyperaceae, popularly known as Priprioca [7]. Our interest in *C. articulatus* L. was based on ethnopharmacological studies that describe its popular use in the Amazon as an analgesic and anti-inflammatory, in addition to other uses [7,8,9]. Several studies have evaluated the pharmacological potential of this species; however, until the present date, we did not find any study reporting the antiproliferative activity of the ethanolic extract of specimens found in the Amazon region.

Analysis of the chemical composition of the essential oil of *C. articulatus* L. reveals the presence of terpene compounds [10]. Sesquiterpenes are known for their anticancer activity [4,6]. During the process to obtain the essential oil from the rhizomes of *C. articulatus* L., a solid residue is generated and discarded. Studies from Kasper et al. [11] on the chemical composition of solid waste extracts revels the presence of sesquiterpenes, such as mustakone and coribolone, fatty acids, and steroids. Also, the hexane extract of priprioca solid residue showed toxicity against microcrustacean and better inhibitory activity against human tumors when used in the antiproliferative assay.

In this study, we sought the chemical characterization of the major volatile compounds present in this solid residue, as well as the evaluation of the cytotoxicity, arginase enzyme activity and in vitro antiproliferative properties of the solid residues generated from the extraction of *C. articulatus* L. oil. The antiproliferative activity was assessed in human tumor cell lines.

## 2. Material and Methods

### 2.1. Plant Material

*C. articulatus* L. were collected in the Amazon community of Tabocal/Santarém-PA (−54°43′00.10″ W and −02°37′41.10″ S), BR-163 Highway, Km 23. The species was identified by botanist Dr. Antonio Elielson Sousa da Rocha, from Emilio Goeldi Museum in Pará (MPEG). An exsiccate was deposited in the the MPEG herbarium and registered under the exsiccate number MG: 207174 in Belém-PA.

### 2.2. Preparation of Ethanolic Extract from Cyperus articulatus L.

3 kg of rhizomes from *C. articulatus* L. were collected, cleaned, ground and dried in open air for three consecutive days. After the drying period, the essential oil was extracted by hydrodistillation for 4h in a 150 L vat. From the vegetable solid waste, obtained from the extraction, 40 g portions were submitted to extraction via Soxhlet using 96% deodorized ethanol. The solution derived from the hot extraction was evaporated in a rotatory evaporator under reduced pressure to obtain a concentrated ethanolic extract of the rhizome of *C. articulatus* L. (EECA). The extraction yields were calculated as a function of the mass of the product obtained as a result of the vegetable mass extracted multiplied by 100 [12].

### 2.3. Chromatographic Analysis of EECA

Analysis of the chemical composition of volatile compounds from the ethanolic extract was performed on a gas chromatograph Agilent model HP-6890 equipped with a selective detector of masses Agilent HP-5975 using a capillary column HP-5MS (30 m × 0.25 mm × 0.25 μm) under the following conditions: injector temperature = 250 °C, column = 80 °C, heating rate 5 °C/min up to 280 °C (20 min) and detector = 300 °C. Helium was used as a carrier gas at a flow rate of 1 mL/min with a selective mass detector operating at 70 eV, m/z = 30 to 500 UMA. EECA was solubilized in ethyl acetate in a concentration of 20 mg/mL and identification of major compounds of the extract was performed by comparison with the electronic library of the equipment (NIST-11).

### 2.4. Mice

C57BL/6 mice (eight weeks of age) were obtained from the UFMG breeding laboratory. The animals were maintained in light/dark cycles of 12 h at a controlled temperature (23 ± 2 °C). Food and water were offered *ad libitum*. The experiments were approved by the committee on the use of animals for experimentation (CEUA/UFOPA) with protocol no. 07004/2013.

### 2.5. Murine Macrophages

Murine Macrophages were obtained by washing the peritoneum of 6 male C57BL/6 mice with sterile frozen PBS, as previously described [13]. Cells were cultured for a period of 24 h and 48 h for evaluation of Cell Viability, and determination of arginase enzyme activity.

### 2.6. Chemicals and Reagents

The following drugs and solutions were used in this study: EECA, Zymosan (RD Systems), thioglycolate (3%), Triton X-100 (Sigma-Aldrich), L-arginine (Merck; pH 9.7), Griess reagent and sodium nitrite (Sigma), MTT (Sigma), α-isonitrosopropiophenone (Sigma), Tris-HCl (Merck), dextran, *Escherichia coli* lipopolysaccharide (Sigma), ethylenediaminetetraacetic acid (sodium EDTA), Sulforhodamine B (Sigma), Doxorubicin hydrochloride (Europharma-SP), urea (Sigma-Aldrich), interferon-γ (Preprotech), dimethyl sulfoxide (DMSO, Fisher Chemical, USA), Luminol (Sigma), Turk’s solution and 96% ethanol (Sigma).

### 2.7. Analysis of Cell Viability

The MTT modified method [14] was used for cell viability analysis. Macrophage cultures maintained for 24 h, stimulated by Lipopolysaccharide (LPS) + interferon-γ (IFN-γ) (1 μg/mL) and zymosan (1 × 10^7^ particles/30 μL) pretreated or not with EECA 1 h before at the concentrations 12.5, 25 and 50 μg/mL, before were washed with PBS buffer (pH: 7.4). The resulting colors were evaluated by spectrophotometric reading at a wavelength of 590 nm. All samples were tested in triplicate.

### 2.8. Arginase Activity

To evaluate the inhibitory effect of EECA in the activity of arginase enzyme in peritoneal murine macrophages stimulated by LPS + IFN-γ (1 μg/mL), the method of Corraliza [15] was used. The cells were pretreated or not with EECA, 1 h before, at concentrations of 12.5, 25 and 50 μg/mL. Arginase activity was measured in cell lysates. Briefly, cells were lysed with 50 µL of 0.1% Triton X-100 (Sigma Chemicals Co., St. Louis, MI, USA). After 30 min on a shaker, 50 µL of 10 mM MnCl_2_ (Merck) and 50 µL of mM Tris HCl (pH 7.5) were added, and the enzyme was activated by heating for 10 min at 55 °C. Arginine hydrolysis was conducted by incubating 25 μL of the activated lysate with 25 μL of 0.5 mM _L_-arginine (Merck) (pH 9.7) at 37 °C for 60 min. The reaction was stopped with 400 μL of (H_2_SO_4_ (96%) H_3_PO_4_ (85%) H_2_O (1/3/7, *v*/*v*/*v*). As a degree of arginase activity, the urea concentration was measured at 540 nm after addition of 25 μL of α-isonitrosopropiophenone (Sigma Chemical Co.) 9% dissolved in ethanol, for 45 min at 95 °C. All samples were tested in triplicate. One unit of arginase activity was defined as the amount of enzyme that catalysed the formation of 1 µmol urea/min).

### 2.9. Antiproliferative Assay

The in vitro antiproliferative activity assay was performed as described by Monks [16]. Human tumor cell lines U251 (glioblastoma, CNS), MCF7 (breast adenocarcinoma), NCI-H460 (lung carcinoma, non-small cells) were used to perform in vitro screening. These cell lines, provided by the National Cancer Institute of the United States (NCI/USA). Samples were also evaluated against one spontaneously transformed human keratinocytes from histologically normal skin (HaCaT cell line) kindly provided by Prof. Dr. Ricardo Della Coletta (University of Campinas, UNICAMP).

Stock cultures were grown in 5 mL of RPMI-1640 supplemented with 5% fetal bovine serum (RPMI/FBS 5%) in a 5% CO_2_ atmosphere at 37 °C and humid environment. A mixture of penicillin and streptomycin (1 μg/mL:1 IU/mL) was added to the experimental cultures. The cells (100 μL of cell suspension per compartment, inoculation density between 3 × 104 and 6.5 × 104 cell/mL) were exposed to different concentrations of EECA (0.25, 2.5, 25 and 250 μg/mL, samples dissolved in DMSO/RPMI/FCS 5%) and Doxorubicin (positive control) in same concentrations and incubated at 37 °C, 5% of CO_2_ in a humid environment for 48 h. The final concentration of DMSO (0.25%) did not affect cell viability. All samples were tested in triplicate.

Cells were fixed with 50% trichloroacetic acid and cell proliferation was determined by spectrophotometric analysis (540 nm) of the cellular protein content using the sulforhodamine B test. Three measurements were obtained at the beginning of incubation (time zero, T0) and 48 h after incubation for compound-free (C) and tested (T) cells. The cell proliferation was determined according to the equation 100 × [(T − T0)/C − T0], for T0 < T < C, and 100 × [(T − T0)/T0], for T ≤ T0 and a concentration-response curve for each cell line was plotted. The GI50 (concentration that produces 50% cell growth inhibition or cytostatic effect) and the TGI (concentration that resulted in total cellular growth inhibition) values were determined from non-linear regression applied to a sigmoidal curve computed by using GraphPad Prism^®^ 6.0 software.

### 2.10. Statistical Analysis

Statistical analysis was performed using the software GraphPad Prim 5.0 (Prim software, Irvine, CA, USA). The results are expressed as the average +/− standard deviation (SD) of at least three different experiments. Analysis of variance (ANOVA two-way) followed by either the Tukey test (T) or Dunnett’s test were applied to evaluate the statistical significance of differences between the study groups. A *p* value < 0.05 was chosen as the criterion for statistical significance.

## 3. Results

### 3.1. Analysis of Chemical Composition of Volatile Compounds of EECA

The yield of the EECA was 7%. GC-MS analysis of the chemical composition of volatile compounds of EECA revealed the presence of eight major compounds, three of which appear in higher concentrations: isocorymbolone, 7-isopropenyl-1,4a-dimethyl-4, 4a,5,6,7,8-hexahydro-2(3H)-naphthalenone, and mustakone (Table 1). It is important to note that (i) the percentages of the compounds listed at Table 1 are related to the total of compounds identified in the GC-MS analysis and not the percentage of the compound in the extract; and that (ii) the GC-MS analysis identify only volatile compounds. 

### 3.2. ECCA Induces Proliferation of Peritoneal Macrophages

**Figure 1:**
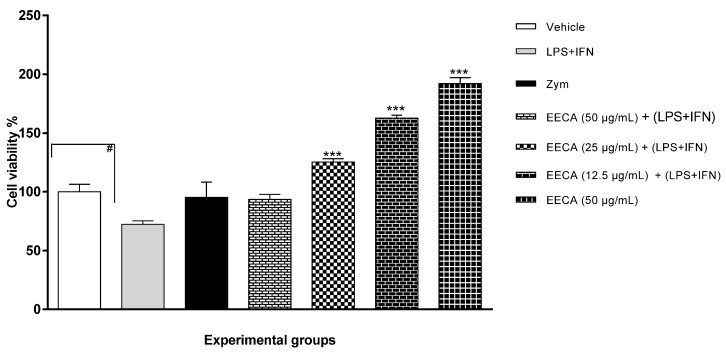
The effect of EECA on the cell viability of peritoneal macrophages stimulated by LPS + IFN-γ and zymosan. Data were obtained from at least three independent experiments and are summarized as mean ± SD. *** *p* < 0.001 indicates a significant difference between the group stimulated by LPS + IFN-γ (1 μg/mL) and treated with EECA. # *p* < 0.1 represents a significant difference between the control group and the group stimulated by LPS + IFN-γ (1 μg/mL). EECA: ethanolic extract of the rhizome of *C. articulatus*.

The results in percentage form show that EECA at concentrations of 12.5, 25 and 50 µg/mL did not affect, in any significant way, the viability of peritoneal macrophages stimulated by LPS + IFN-γ (1 μg/mL) and zymosan (1 × 10^7^ particles/30 μL), since the percentage of cell viability of the groups treated with EECA exceeded 100% (Figure 1).

The results obtained demonstrate that the concentrations of EECA tested did not negatively affect the viability of macrophages (*p* < 0.0001). In addition, there is an increase in the metabolic activity of these cells, as measured by the increase of formation of formazan crystals in MTT, when exposed to the concentrations of the extract (EECA 25 and 50 (µg/mL) + LPS + IFN-γ (1 μg/mL)) and (EECA 50 (µg/mL)), with the exception of 12.5 µg/mL (*p* < 0.0001).

In the cell viability test we observed that there was efficiency in the applied methodology, as the stimulus group (LPS + IFN-γ 1 μg/mL) had a negative effect (−27.8%) on the percentage of viable cells. The group that received Zymosan also presented a reduction (−5%) in the percentage of viability. The groups that were treated with EECA did not present a significant reduction in the percentage of viable cells; even in the group with the highest concentration of EECA, the reduction was 192%.

### 3.3. Arginase Acttivity (Murine Macrophages)

**Figure 2:**
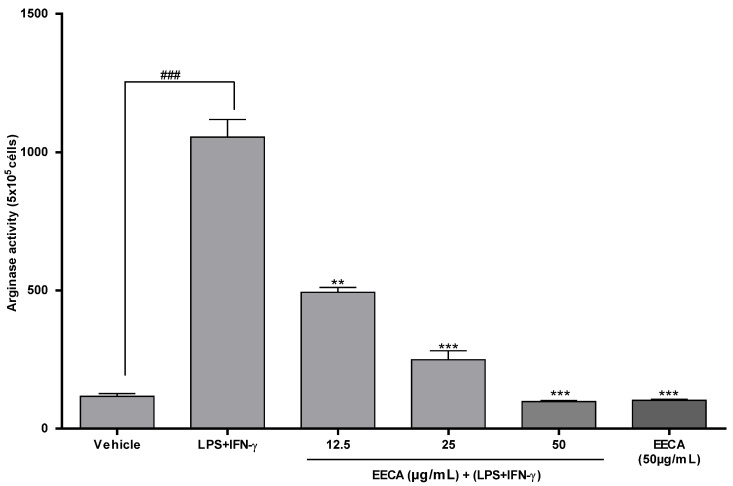
The effect of EECA on the activity of the arginase enzyme in IFN-γ stimulated peritoneal macrophages and LPS + IFN-γ. Data were obtained from at least three independent experiments and are expressed as mean ± SD. ** *p* < 0.01 and *** *p* < 0.001 indicate a difference between the LPS + IFN-γ (1 μg/mL)-stimulated group and the EECA-treated group. ^###^ *p* < 0.001 indicates a significant difference between the control group (mean + 2% of DMSO) and the group stimulated by LPS + IFN-γ (1 μg/mL). EECA: ethanolic extract of the rhizome of *C. articulatus*.

The results of this in vitro experimental model demonstrated that treatment with EECA at concentrations of 12.5, 25 and 50 µg/mL reduces in a statistically significant way the activity of arginase in peritoneal macrophages stimulated by LPS + IFN-γ (1 μg/mL), (*p* < 0.0001) (Figure 2).

We observed that the proposed experimental protocol was viable, that stimulation with LPS + IFN-γ (1 μg/mL) promoted an 896.7% increase in the activity levels of the arginase enzyme. The results observed in the groups treated with EECA showed a reduction in this activity, and even when in the concentration of 12.5 μg/mL the reduction was 53.2% and in the concentration of 25 μg/mL the reduction was 76.3%; in the highest concentration tested (50 μg/mL), the percentage of reduction in relation to the only stimulated group was 90.7%.

### 3.4. In Vitro Antiproliferative Activity (Human Tumor Cell Lines)

The antiproliferative activity presented a statistically significant influence of the cell line type and the concentrations of EECA tested (Concentrations EECA vs. Cellular lineages) (*p* < 0.0001). The Table 2 presents the GI_50_ and TGI values of the EECA in vitro antiproliferative activity. 

The EECA in the concentrations used showed a significant reduction effect on the proliferation of the different cell lines (*p* < 0.0001). At the lowest concentration, which corresponds to 0.001 μg/mL of EECA, there was no significant difference in relation to inhibition of cell proliferation of the different cell lines. In turn, there was a significant reduction in cell proliferation in the 0.250 and 2.5 µg/mL of EECA, only in lineage NCI-H460 in relation to the HaCat cell line when exposed to the same extract concentration (*p* < 0.0001).

**Figure 3:**
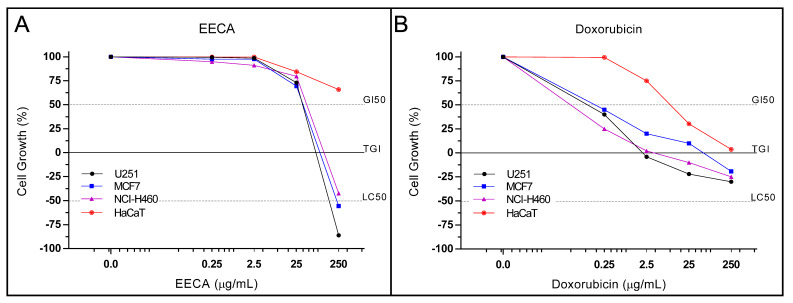
(**A**) Cell proliferation curve as a function of the EECA concentration dissolved in a DMSO/RPMI/5% FCS medium. (**B**) Cell proliferation curve as a function of Doxorubicin concentration. The effective concentration resulting in TGI (total growth inhibition, concentration required for a total inhibition of cell proliferation) was calculated by sigmoidal, nonlinear regression. GI50: concentration that produces 50% cell growth inhibition or cytostatic effect; TGI: concentration that resulted in total cellular growth inhibition; LC50: concentration that produces 50% cell death or cytotoxic effect. EECA: ethanolic extract of the rhizome of *C. articulatus*.

At the highest concentrations tested, corresponding to 25 and 250 µg/mL of EECA, there was a significant reduction in cell proliferation in all tested cell lines (NCI-H460, U251 e MCF7) in relation to the HaCat cell line when exposed to the same extract concentration (<0.0001) (Figure 3).

EECA in concentrations above 25 µg/mL inhibited growth of glioblastoma U251, breast adenocarcinoma MCF7 and lung carcinoma, non-small cells NCI-H460 tumor cells. Furthermore, the value of Total Growth Inhibition (TGI) in antiproliferative activity for the extract was 62.7 µg/mL acting on glioblastoma U251 tumor cells. For the other tumor cells such as breast adenocarcinoma MCF7 and lung carcinoma, non-small cells NCI-H460, the TGI for ethanolic extract was 100 and 135.7 µg/mL (Figure 3).

## 4. Discussion

It has been previously demonstrated that the essential oil of *Cyperus articulatus* L. is mainly constituted by different sesquiterpenes structures, such as caryophyllene, eudesmane, patchoulane and rotundane [17]. Our study was focused on the solid residue generated after the process of obtaining essential oil from the rhizomes of *C. articulatus* L. Chemical analysis of volatile compounds from this residue revealed the presence of sesquiterpenes that are also present in the essential oil of the species, indicating that the hydrodistillation process was not fully efficient due to the nature of the extracted material (residue). One limitation of the present study was the chemical analytical method, since the GC-MS only identify volatile compounds, some of which are lost in the extract preparation, being a most appropriate methodthe HPLC, UPLC or other liquid-phase chromatography. However, it is necessary to clarify that after the distillation of the essential oil, some volatile sesquiterpenes are still present in the solid residue and therefore must be detected by GC-MS. Considering previous studies from our group, we understand that there are still important volatile substances in the ethanolic extract, and these groups of substances are associated with antiproliferative activity [11]. Many of the biological activities cited for *C. articulatus* L. are attributed to sesquiterpenes present in this species. However, it is worth noting that, to date, no studies have been found on the chemical composition of extracts obtained from solid residues derived from the extraction of essential oil from *C. articulatus* L., the object of our study. In addition, we also evaluated the biological activity of EECA regarding its potential ability to intervene in the cell proliferation of some cancer cell lines. 

Although significant fractions of the volatile compounds were removed by hydrodistillation during the extract preparation, we were able to identify eight different substances through GC-. The isocorimbolone was found to be the volatile compound most abundant, followed by mustakone and (7-isopropenyl-1,4a-dimethyl-4,4a, 5,6,7,8-hexa -hydro-2 (3H) -naphthalenone. Studies of the composition of hexane extract of *C. articulatus* L., collected in the Republic of Cameroon, showed the presence of corymbolone, α-corymbolol, mandassidione, mustakone, α-cyperone, and isopatchoulenone [18] close to the results obtained from EECA in our study. 

It has been shown that the chemical composition of *Cyperus articulatus* L. contains substances such as mustakone and corimbolone, the latter being the main substance of the extract [17]. The results from chemical analysis of the volatile compounds are in accordance with the previous literature, where corimbolone and mustakone were identified among the main substances. Cyperotundone, which was identified in the extract of *C. articulatus* L. [17] was also found in EECA in relatively low proportions. Other studies describe the identification of cypertrogone in the essential oil of other *C.* species, such as *C. papyrus* L. [19] and *C. rotundus* L. [20]. Mustakone, the major compound of the essential oil of *C. articulatus*, also identified in the EECA, showed antipasmodic and antinematodal activities. [21,22] Our results showed that the EECA has no cytotoxicity in concentrations lower than 50 µg/mL, which is consistent with the literature indicating that concentrations around 50 µg/mL of aromatic herbal extracts show no cytotoxic action [8]. The EECAat a concentration of up to 50 µg/mL, did not had cytotoxicity in peritoneal macrophages and presented cytoprotective characteristics, increasing the percentage of viability of cultured cells, evidenced by increased production of formazan crystals from macrophages (Figure 1). 

In this work we also evaluated the effect of EECA on the activity of the enzyme arginase, and we know that this enzyme in addition to its physiological actions is also related to pathological processes such as cancer and inflammation. Cell proliferation is a process mediated by action of type 2 arginase, an inducible enzyme, which when activated catalyzes the biosynthesis of the polyamines glutamate and proline [23].

According to our results, this enzyme suffers a reduction in its activity by action of EECA. Other studies report finding two to three-fold higher polyamine levels in breast cancer tissues compared to healthy tissues [24,25]. The activity of the arginase enzyme undergoes a reduction in the presence of the EECA in the concentrations tested, since it aligns with the test of the cell proliferation of tumor lines. In our research, we used known cell lines that allowed an adequate evaluation of the action of EECA on these cells. Here we used three different lineages - glioblastoma (U251), breast (MCF7) and lung (NCI-H460) [26]. 

Drugs used in chemotherapy are able to induce cytotoxicity in tumor cells by various mechanisms, especially by acting on cell signaling [27]. Currently there is a great need to look for new anticancer drugs with the ability to perform selective cytotoxicity on cancer cells with low toxicity in healthy cells [28]. Natural products from plants can be a source of molecules with antiproliferative activity, with several recent reports worldwide [29,30,31,32,33]. In our study the EECA was first tested against human tumor lines. We observed that in concentrations over 10^2^ µg/mL the extract inhibited cell growth of glioblastoma U251 tumor cells, breast adenocarcinoma MCF7 and non-small cell lung carcinoma, NCI-H460 without present toxicity to HaCat. However, in glioblastoma U251 tumor cells the antiproliferative activity was highest with a TGI value of 62.7 µg/mL. Our results resemble the study of Mazzio et al. [34], which demonstrated that ethanolic extract of rhizomes of *C. rotundus* showed moderate anticancer activity in Neuro-2a cells (LC50 = 2.528 to 4.939 mg/mL calculated from dose-dependent cell death). Riva [35] tested the ethanolic extract of tubers of *S. allagophylla* against eight tumor cell lines, which presented a weak in vitro antiproliferative effect, except for the line of K-562 cells (leukemia), whose growth was completely inhibited with TGI concentration of 5.13 μg/mL (Figure 3). In summary, the major compounds identified in EECA belong to the sesquiterpenes group (e.g., isocorimbolone and mustakone) and demonstrated significant anti-proliferative activity against the tumor cell lines tested.

## 5. Conclusions

The production of essential oil from the rhizomes of the Amazonia plant *C. articulatus* resulted in large amounts of residue production, considered as waste. Our study showed that this residue still has volatile biologically active substances, primarily sesquiterpenes, and that the ethanolic extract of the residue reduced the proliferation of cancer cells. The results indicated that the extract of *C. articulatus* residue exerts antiproliferative activity with low cytostatic potency on healthy cells in experimental models in vitro. Further studies with fractions of the EECA and isolation of specific sesquiterpenes are required to elucidate which molecule has the antiproliferative properties or if the results were from synergistic associations between sesquiterpenes and/or other compounds not yet identified. 

## Figures and Tables

**Table 1:** Major constituents identified through GC-MS and their respective concentrations found in ethanolic extract of the rhizome of *Cyperus articulatus* (EECA).

t_R_ (min)	Identification	% *
24.22	Isocorimbolone	25.38
19.88	Mustakone	23.89
21.43	7-isopropenil-1,4a-dimetil-4,4a,5,6,7,8-hexahidro-2(3H)-naftalenona	18.40
23.56	Corimbolone	10.86
20.53	Aristolone	6.69
22.51	Mandassidione	5.76
20.30	Cyperontundone	5.48
6.29	Undecan	3.54

t_R_ = Retention time (minutes); % = Relative percentage (in relation to total compound found in GC-Ms analysis), * Percentages were calculated only in relation to majority volatile compounds identified.

**Table 2:** GI50 and TGI values for extracts of essential oil of *C. articulatus*.

Cell Lines	EECA GI_50_ (µg/mL)	EECA TGI (µg/mL)
U251 (*human tumor cell lines*)	37.29	627
MCF7 *(human breast adenocarcinoma)*	36.78	100
NCI-H460 *(human lung carcinoma)*	41.51	135
HaCat *(human normal skin cell)*	26.72	>250

GI_50_ (concentration that produces 50% cell growth inhibition or cytostatic effect) and TGI (concentration that resulted in total cellular growth inhibition). EECA: ethanolic extract of the rhizome of *C. articulatus*.

## Data Availability

All data, tables and figures in this manuscript are original. Raw data of this experiment is fully evaluable upon request to the corresponding author.
